# Probiotics for preventing and treating infant regurgitation: A systematic review and meta‐analysis

**DOI:** 10.1111/mcn.13290

**Published:** 2021-12-15

**Authors:** Jann P. Foster, Hannah G. Dahlen, Sabina Fijan, Nadia Badawi, Virginia Schmied, Charlene Thornton, Caroline Smith, Kim Psaila

**Affiliations:** ^1^ School of Nursing and Midwifery Western Sydney University Penrith New South Wales Australia; ^2^ Ingham Research Institute Liverpool New South Wales Australia; ^3^ New South Wales Centre for Evidence Based Health Care: A JBI Affiliated Group Penrith New South Wales Australia; ^4^ Faculty of Health Sciences University of Maribor Maribor Slovenia; ^5^ Grace Centre for Newborn Intensive Care The Children's Hospital at Westmead Westmead New South Wales Australia; ^6^ Cerebral Palsy Alliance Research Institute University of Sydney Camperdown New South Wales Australia; ^7^ The National Institute of Complementary Medicine Western Sydney University Penrith New South Wales Australia

**Keywords:** infant, infant regurgitation, probiotic, reflux

## Abstract

Infant regurgitation is common during infancy and can cause substantial parental distress. Regurgitation can lead to parental perception that their infant is in pain. Parents often present in general practitioner surgeries, community baby clinics and accident and emergency departments which can lead to financial burden on parents and the health care system. Probiotics are increasingly reported to have therapeutic effects for preventing and treating infant regurgitation. The objective of this systematic review and meta‐analysis was to evaluate the efficacy of probiotic supplementation for the prevention and treatment of infant regurgitation. Literature searches were conducted using MEDLINE, CINAHL, and the Cochrane Central Register of Controlled trials. Only randomised controlled trials (RCTs) were included. A meta‐analysis was performed using the Cochrane Collaboration methodology where possible. Six RCTs examined the prevention or treatment with probiotics on infant regurgitation. A meta‐analysis of three studies showed a statistically significant reduction in regurgitation episodes for the probiotic group compared to the placebo group (mean difference [MD]: −1.79 episodes/day: 95% confidence interval [CI]: −3.30 to −0.27, *N* = 560), but there was high heterogeneity (96%). Meta‐analysis of two studies found a statistically significant increased number of stools per day in the probiotic group compared to the placebo group at 1 month of age (MD: 1.36, 95% CI: 0.99 to 1.73, *N* = 488), with moderate heterogeneity (69%). Meta‐analysis of two studies showed no statistical difference in body weight between the two groups (MD: −91.88 g, 95% CI: 258.40–74.63: *I* ^2^ = 23%, *N* = 112) with minimal heterogeneity 23%. Probiotic therapy appears promising for infant regurgitation with some evidence of benefit, but most studies are small and there was relatively high heterogeneity. The use of probiotics could potentially be a noninvasive, safe, cost effective, and preventative positive health strategy for both women and their babies. Further robust, well controlled RCTs examining the effect of probiotics for infant regurgitation are warranted.

## Key messages

- Infant regurgitation is common during infancy and can cause substantial parental distress.
- The currently available evidence does not support or refute the efficacy of probiotics for the prevention and treatment of infant regurgitation but data from the individual trials and subset meta‐analysis of studies are promising.
- There are no indications from the available data that probiotics have any adverse effects.
- Further well‐controlled RCTs are warranted to investigate the efficacy of various strains and species, dosage, and combinations of probiotics.

## Graphical abstract

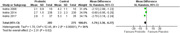

## 1 BACKGROUND

The European Society for Pediatric Gastroenterology, Hepatology, and Nutrition Guidelines defines gastro‐esophageal reflux as the passage of gastric contents into the esophagus, with or without regurgitation and vomiting (Rosen et al., 2018). Reflux is the most common functional gastrointestinal disorder in the first year of life (Van Tilburg et al., 2015). The natural progression of reflux in infants is between birth and 4 months of age and normally decreases significantly by 12 months of age (Baird et al., 2015; Davies et al., 2015; Hegar et al., 2009) and is not associated with negative long‐term consequences. The prevalence of reflux between 2 and 4 months of age is approximately 40% of infants (Baird et al., 2015; Campanozzi et al., 2009; Martin et al., 2002; The Royal Children's Hospital Melbourne, 2019).

When the gastro‐esophageal reflux is high enough for the stomach contents to be visualised coming out of the infant's mouth, it is called regurgitation (Benninga et al., 2016; Rosen et al., 2018). The diagnosis of infant regurgitation is primarily based on the symptom‐based Rome III and Rome IV criteria. In 2016, the Rome criteria for functional gastrointestinal disorders were revised for infants/toddlers. No changes were made for infant regurgitation in the updated Rome IV compared to the Rome III (Benninga et al., 2016; Hyman et al., 2006). The Rome Diagnostic Criteria for Infant Regurgitation must include both of the following in otherwise healthy infants 3 weeks to 12 months of age:

1. Regurgitation two or more times per day for 3 or more weeks.
2. No retching, hematemesis, aspiration, apnea, failure to thrive, feeding or swallowing difficulties, or abnormal posturing, and no other signs should be present (Benninga et al., 2016).

Conversely, gastro‐oesophageal reflux disease (GORD) refers to reflux that causes troublesome symptoms and infant distress, with or without complications such as damage to the oesophagus (Vandenplas & Rudolph, 2009).

Regurgitation may occur after feeding more than six times per day in some infants (Anabrees et al., 2013; Ferguson, 2018) and can cause substantial anxiety in parents. They may also perceive their infant's crying as being due to pain (Walls, 2019). As a result, most often parental concern is the factor driving the push for a diagnosis and treatment (Davies et al., 2015; Rosen et al., 2018). Indeed, it has been estimated that during the first 6 months after birth, approximately 25% of appointments with paediatricians and other health professionals are due to infant regurgitation and treatment (Francavilla et al., 2015). There can be significant personal and public health care expenses, because of professional consultation fees, over‐the‐counter medication or home remedies, use of special milk formulas, and loss of income due to work absenteeism (Salvatore et al., 2018).

Medical interventions are not required for postprandial regurgitation (Zeevenhooven et al., 2017) and should primarily be managed with reassurance, education, and support for parents (Walls, 2019; Zeevenhooven et al., 2017) and avoidance of medication (Walls, 2019). Conservative treatments include thickened feeds, antiregurgitation thickened formulas, and postprandial positioning. However, there is currently a lack of evidence on the effectiveness, or safety to support these commonly recommended nonpharmacological conservative management strategies for regurgitation (Bell et al., 2018; Dahlen et al., 2018). A recent Cochrane review (Kwok et al., 2017) found moderate quality of evidence that feed thickeners for formula fed infants reduce the number of regurgitations by nearly two episodes per day (mean difference [MD] − 1.97, 95% CI [confidence interval]: −2.32 to −1.61, 6 studies and 442 infants). However, feed thickeners may increase caloric density, and the long‐term impact of providing infants with such high carbohydrate and low protein feed is unclear (Kwok et al., 2018) and are not an option for direct breastfeeding mothers.

In addition, prominent researchers on regurgitation Salvatore, Tabbers, Singendonk, Savino, Staiano, Benninga, Huysentruyt, and Vandenplas argue that positional management of infants with regurgitation (side sleeping or elevated supine position) cannot be recommended in sleeping infants due to insufficient evidence regarding efficacy and safety (Salvatore et al., 2017). Probiotics are increasingly being proposed as a possible conservative therapeutic strategy with minimal side effects that may help to modify regurgitation symptoms. Probiotics are defined as live micro‐organisms that, when administered in adequate amounts, confer a health benefit on the host (Hill et al., 2014). The intestinal microbiota plays a crucial role in the pathogenesis of gastrointestinal disorders (Di Mauro et al., 2013; Indrio et al., 2014) and an increasing number of studies are targeting probiotic therapy (Al Faleh & Anabrees, 2014; Allen et al., 2010; Anabrees et al., 2013; Baldassarre et al., 2016; Chau et al., 2015; Di Mauro et al., 2013; Goldenberg et al., 2017; Hoveyda et al., 2009; Newlove‐Delgado et al., 2017; Savino et al., 2010; Sung et al., 2013; Szajewska et al., 2013) for infants and adults. The most used probiotics in these studies are certain strains or species of lactobacilli and bifidobacteria, such as *Lactobacillus rhamnosus* GG, *Lactobacillus reuteri* DSM 17938, *Lactobacillus reuteri* ATCC 55730, *Lactobacillus casei Shirota*, *Lactobacillus acidophilus*, *Lactobacillus bulgaricus*, *Lactobacillus plantarum* 299v, *Bifidobacterium lactis* BB12, *Bifidobacterium breve Yakult*, and the yeast *Saccharomyces boulardii*.

The pathophysiology of functional regurgitation is still controversial and seems to be multifactorial (Indrio et al., 2011) but there is growing evidence that an abnormal gut microbiota colonisation may play a crucial role (Indrio et al., 2014). An early probiotic supplementation may alter colonisation and represent a new strategy for preventing functional gastrointestinal disorders. Mechanisms of action include enhanced epithelial barrier, inhibition of mucosal pathogens and increased adhesion of favourable micro‐organisms to the intestinal mucosa, and production of antimicrobial substances (bacteriocins, acids, etc.) and immune system modulation (Bermudez‐Brito et al., 2012; Hemaiswarya et al., 2013). All this is done by manipulation of the human microbiome, especially the intestinal microbiota (Gilbert et al., 2018). It is also noted that probiotics could play a role in controlling intestinal inflammation (Indrio et al., 2014, 2017). In addition, gastric distension and impaired fundal relaxation due to disturbed gastric motility might be a contributor to infant regurgitation (Indrio et al., 2011). Probiotics are reported to mediate the activity on colonic sensory neurons, specifically the calcium‐dependent potassium ion channel in enteric sensory nerves, resulting in an improvement in gut motility and gastric emptying time and effects on visceral pain (Collins & Bercik, 2009; Garofoli et al., 2014; Wang et al., 2010). This indicates that there are potential mechanisms for the benefits of probiotics in infant regurgitation.

Several systematic reviews have found probiotics to be effective for small intestinal bacterial overgrowth in children with regurgitation treated with probiotics and proton pump inhibitors (Belei et al., 2018), necrotizing enterocolitis in preterm infants (Al Faleh & Anabrees, 2014) and regurgitation in adults (Cheng & Ouwehand, 2020). A systematic review (Sung et al., 2013) concluded that probiotics may be an effective treatment for breastfed infants with colic. However, a more recent Cochrane review found no clear evidence to support the use of probiotics for infantile colic (Ong et al., 2019). A recent prospective observational study reported that, since the introduction of probiotics into a neonatal intensive care unit, the exclusive use of omeprazole, a proton‐pump inhibitor that decreases the amount of acid production in the stomach, had dropped from 51.6% to 24% (*p* = 0.01) in preterm infants (Deshpande & Pawar, 2019). We were not able to identify any systematic reviews on the use of probiotics for the treatment or prevention of infant regurgitation.

## 2 METHODS

The objective of this systematic review was to determine the effectiveness of probiotics for the prevention and treatment of infant regurgitation in term and preterm infants up to 12 months of age following birth. The systematic review followed the methods described in the *Cochrane Handbook for Systematic Reviews of Interventions* and by the Cochrane Neonatal Review Group (Higgins & Green, 2017).

### 2.1 Outcomes

The primary outcome for the infants was the effect of probiotics on episodes of infant regurgitation per day (using Rome III/IV, or as defined by the authors). The secondary outcomes were the effect of probiotics on gastric emptying time, number of stools, growth rate (weight, head circumference, and length), admissions to hospital related to infant regurgitation, loss of parent working days related to infant regurgitation, number of admissions of mother to hospital due to anxiety/depression, number of visits to any health professional, and adverse events related to probiotic supplementation (mother and infant).

### 2.2 Search strategy

Eligible studies were sought from the Cochrane Central Register of Controlled Trials (CENTRAL 2020, Issue 5) in the Cochrane Library; MEDLINE via PubMed (1966 to 9 April 2021); Embase (1980 to 9 April 2021); and CINAHL (1982 to 9 April 2021) using the following subject MeSH headings and text word terms: ‘neonate(s)’, ‘newborn(s)’, ‘infant(s)’, AND ‘regurgitation’ OR ‘infant regurgitation’ OR ‘infantile reflux’ OR ‘reflux’ AND ‘probiotic’. Language restrictions were not applied. We searched clinical trials registries for ongoing or recently completed trials (World Health Organization International Clinical Trials Registry Platform (ICTRP): https://www.who.int/clinical-trials-registry-platform/the-ictrp-search-portal; US National Library of Medicine: clinicaltrials.gov; ISRCTN Registry https://www.isrctn.com/). All potentially relevant titles and abstracts were identified and retrieved during the search. Independent hand searches were undertaken, and the bibliographies of each article were assessed for additional relevant titles.

### 2.3 Study selection and data extraction

We included randomised controlled trials (RCTs) that compared probiotics (any dose or composition) to placebo, control, or other forms of treatment in mothers during the antenatal period, and term and preterm infants in the postnatal period (from birth and up to 12 months) for the prevention (mother/infant) and treatment (infant) of infant regurgitation. Articles in any language were considered if there was an abstract in English.

We used the data extraction form available within Review Manager software (RevMan) to extract data on the participants, interventions and control(s), and outcomes of each included trial. Two review authors (JF and KP) screened the title and abstract of all identified studies. The titles were also checked by third author (SF). We reassessed the full text of any potentially eligible reports and excluded the studies that did not meet all the inclusion criteria. Two review authors (JF and KP) independently extracted data from each study without blinding to authorship or journal publication. In case of any disagreement, the three review authors resolved them by discussion until reaching a consensus. One review author (JF) entered data into RevMan, and two review authors (KP and SF) verified them (Higgins & Green, 2017).

### 2.4 Methodological quality of the studies

Standard methods of the Cochrane Collaboration as described in The Cochrane Library (www.thecochranelibrary.com) were used to assess the methodological quality of included trial (Higgins & Green, 2017). The methodological details of the studies were extracted from published data. For each trial, information was sought regarding:

- Selection bias: Random sequence generation due to inadequate generation of a randomised sequence and inadequate concealment of allocations before assignment.
- Blinding of participants and personnel: Performance bias due to knowledge of the allocated interventions by participants and personnel during the study.
- Blinding of outcome assessment: detection bias due to knowledge of the allocated interventions by outcome assessors.
- Incomplete outcome data: attrition bias due to amount, nature, or handling of incomplete outcome data.
- Selective reporting: reporting bias due to selective outcome reporting.
- Other sources of bias: bias due to problems not covered elsewhere in the table.

### 2.5 Data synthesis

We performed statistical analyses using Cochrane's Review Manager (Higgins & Green, 2017). We analyzed continuous data using mean differences (MDs) and report the 95% CI on all estimates. We used the random‐effects model for all meta‐analyses and assessed the heterogeneity between the included trials, using the *I* ^2^ statistic. The degree of heterogeneity was graded as nonexistent or minimal for an *I* ^2^ value of less than 25%, low for an *I* ^2^ value of 25%–49%, moderate for an *I* ^2^ value of 50%–74%, and high for an *I* ^2^ value of 75%–100%. We planned to assess sources of heterogeneity using sensitivity and subgroup analysis, however, there were insufficient data.

## 3 RESULTS

**Figure 1:**
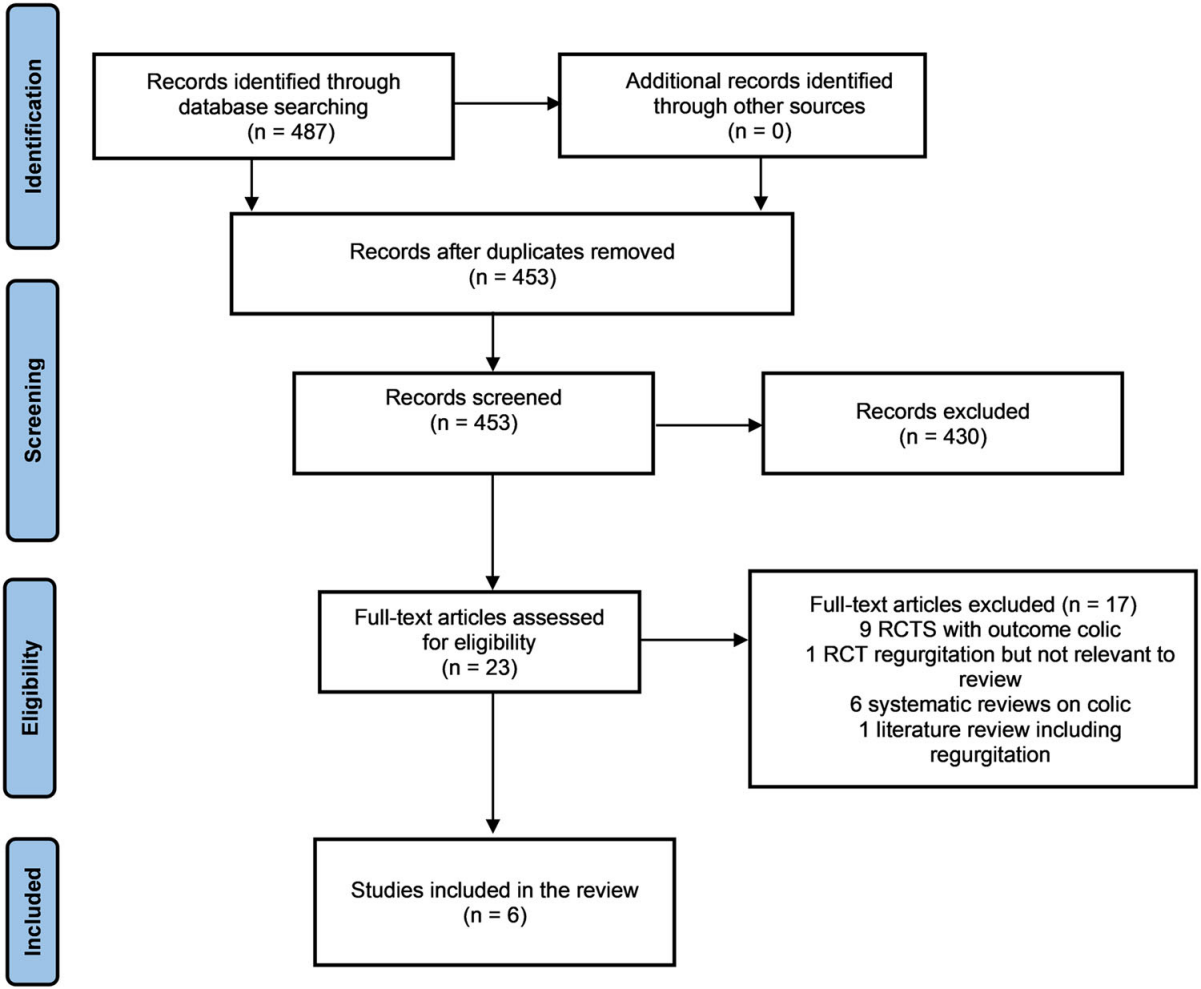
PRISMA study flow diagram

The database searches retrieved 486 titles and abstracts. After removal of duplicates, 452 unique titles remained. A total of 22 potentially relevant citations were obtained through our primary search strategy. Sixteen articles were excluded because they investigated the use of probiotics for infantile colic. Six RCTs met the inclusion criteria (Baldassarre et al., 2016; Garofoli et al., 2014; Indrio et al., 2011, 2014, 2017). No ongoing studies were identified. A flow diagram of the identification and selection of studies is shown in Figure 1.

A total of 736 infants and 67 women (and matching infant) were enroled across the six studies.

Characteristics of the included trials are summarised in Appendix 1. All studies were parallel RCTs and compared probiotics versus placebo for treating (Indrio et al., 2017) or preventing (Baldassarre et al., 2016; Garofoli et al., 2014; Indrio et al. 2008, 2014) infant regurgitation. Infants in one of the studies were preterm (Indrio et al., 2008) and term in the remaining five studies. One study (Baldassarre et al., 2016) administered 99 billion viable lyophilised bacteria that consisted of four different strains of lactobacilli (*L. paracasei* DSM 24733, *L. plantarum* DSM 24730, *L. acidophilus* DSM 24735, *and L. delbrueckii* subsp. *bulgaricus* DSM 24734, three strains of bifidobacteria (*B. longum* DSM 24736, *B. breve* DSM 24732, and *B. infantis* DSM 24737), and one strain of *Streptococcus thermophilus* DSM 24731. The probiotics in this study were given to mothers 4 weeks before the expected delivery date (36th week of pregnancy) until 4 weeks after delivery (Baldassarre et al., 2016). Outcomes were measured at 1‐month postbirth.

The remaining five studies administered *L. reuteri* DSM 17938 or its original strain, *L. reuteri* ATCC 55730 to preterm and formula fed infants (Indrio et al., 2008), full term and formula fed infants (Indrio et al., 2017), full term and formula or breastfed infants (Indrio et al., 2014) or full term and breastfed infants (Garofoli et al., 2014). Garofoli et al. (2014), Indrio et al. (2008), and Indrio et al. (2011) administered 1 × 10^8^ colony‐forming units in 5 drops/day for 28–30 days. Indrio et al. (2014) also administered 1 × 10^8^ colony‐forming units in 5 drops/day for 30 and 90 days. Indrio et al. (2017) used three different antiregurgitation strategies: 2.8 × 10^8^ colony‐forming units/g powder, a partially hydrolysed 100% whey formula and thickened with starchfor 30 days.

Indrio et al. (2011) and Indrio et al. (2017) administered the probiotics to infants already diagnosed with uncomplicated regurgitation and were enroled at 31–45 days and 1–5 months postbirth, respectively. Infants in the remaining three RCTs were enroled in the first week postbirth. Outcomes were measured after the infants received probiotics or placebo for at least 1 month (Garofoli et al., 2014; Indrio et al. 2011, 2017) and 1 and 3 months in the Indrio et al. (2014) study (Appendix 1).

### 3.1 Infant regurgitation

**Figure 2:**
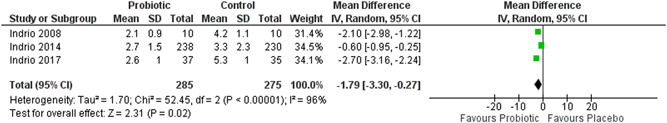
Probiotic versus placebo—regurgitation (no. per day) after 1 month of intervention

Six trials examined the effect of probiotics on episodes of regurgitation per day following 1 month of intervention. Three studies were included in the meta‐analysis (Indrio et al. 2008, 2014, 2017). Meta‐analysis showed a statistically significant reduction in regurgitation in the probiotic group compared to the placebo group (MD: −1.79 episodes/day, 95% CI: −3.30 to −0.27, *N* = 560, *p* = 0.02) (Figure 2). The *I* ^2^ statistic of equal to 96% indicates high heterogeneity.

We were unable to include the remaining three studies in the meta‐analysis due to the method of reporting. Garofoli et al. (2014) reported for infants receiving the probiotic, a significant reduction was shown in the average daily number of regurgitations (*p* = 0.02) from baseline to Day 28 of the study period, but did not provide any summary data. At the end of the second week the difference with the placebo group was significant (*p* = 0.05) and there was a trend to a significant result reported at the end of the third week of treatment (*p* = 0.06). Indrio et al. (2011) found infants receiving the probiotic had a significant decrease in episodes of regurgitations/day compared to placebo: Median 1.0 (5th percentile = 1.0; 95th percentile = 2.0) versus Median 4.0 (5th percentile 3.0; 95th percentile = 5.0), (*p* < 0.001). Baldassarre et al. (2016) reported that the onset of regurgitation was significantly reduced in the probiotic group compared to the placebo group when administered to women 4 weeks before the expected delivery date until 4 weeks after delivery: *χ* ^2^ = 6.944, *p* = 0.008; relative risk = 2.43 (95% CI: 1.14–5.62).

Indrio et al. (2014) was the only study to report regurgitation after 3 months of the commencement of the intervention and found a statistically significant reduction in regurgitation for infants receiving the probiotic compared to the placebo (MD: −1.70 episodes/day: 95% CI: −2.14 to −1.26, *N* = 468, *p* = 0.00001).

### 3.2 Gastric emptying time

Due to the method of reporting for gastric emptying time, we were unable to perform meta‐analysis. Indrio et al. (2008) reported that gastric emptying rate (%) was statistically significantly faster in the newborns receiving probiotics compared with a placebo (25% vs. 50%, *p* < 0.001). Indrio et al. (2011) reported the change in gastric emptying rate (%) before and after the intervention and found a statistically significantly increased gastric emptying rate in infants receiving probiotics compared to placebo +11.7 (−3.9 to +24.0)% versus +8.4 (−27.0 to +23.5)%, *p* = 0.01. Indrio et al. (2017) also reported a significantly increased gastric emptying rate percentage change for infants receiving probiotics: median 12.3 (5th percentile = −3.9, 95th percentile = 22.0) compared to placebo: Median 9.1 (5th percentile = −27.0; 95th percentile = 25.5), *p* < 0.01.

### 3.3 Number of stools

**Figure 3:**
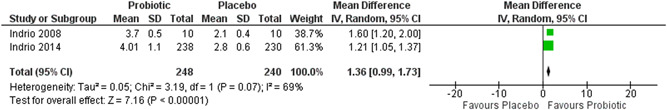
Probiotic versus placebo—no. stools per day after 1 month of intervention

Meta‐analysis of two studies (Indrio, 2014, 2008) found a statistically significant increase in the number per day of stool evacuations in the probiotic group compared to the placebo group at 1 month (MD: 1.36, 95% CI: 0.99 to 1.73, *N* = 488, *p* = 0.00001). However, the *I* ^2^ statistic of equal to 69% indicates moderate heterogeneity (Figure 3). We were unable to include the remaining two studies in the meta‐analysis due to the method of reporting. Baldassarre et al. (2016) reported no significant differences between the probiotic and placebo groups in number of bowel movements at 1 month (3.7 vs. 4.2, *t* = 1.17, *p* = 0.246). Garofoli et al. (2014) reported 'similar pattern for the probiotic and placebo groups in the daily stool frequency' at 1 month, but no data was provided.

Only one study reported no. of stools/day after 3 months' administration of the probiotic/placebo. Indrio et al. (2014) and found a significant increase in the probiotic group compared to the placebo group (MD: 0.60 stools/day, 95% CI: 0.27–0.93, *N* = 468, *p* = 0.0003).

### 3.4 Growth (body weight, head circumference, and length)

**Figure 4:**
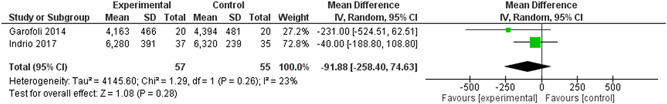
Probiotic versus placebo—body weight after 1 month of intervention

Four studies reported on total body weight after 1 months' administration of the probiotic/placebo. Two studies were able to be included in the meta‐analysis (Garofoli et al., 2014; Indrio et al., 2017). No statistical difference was found between the probiotic and placebo groups (MD: −91.88 g, 95% CI: 258.40–74.63: *I* ^2^ = 23%, *N* = 112, *p* = 0.28] (Figure 4). We were unable to include the remaining two studies in the meta‐analysis due to the method of reporting. Indrio et al. (2011) reported no difference in body weight between the two groups during, and at the end of the trial, but no data was provided. Indrio et al. (2008) reported no difference in weight gain in grams per day over the last 7 days of 1 months' treatment between the probiotic and placebo groups (MD: 3 g/day, 95% CI: −3.64 to 9.64, *N* = 20, *p* = 0.38).

Garofoli et al. (2014) reported no difference in baby length between the probiotic group (55.1 cm, *SD*: 1.94) and control group (56.45, *SD*: 0.65), *p* = 0.087 at 4 weeks after commencing treatment. However, when calculating summary data, we found a significant different between the two groups (−1.35 cm [−2.25 to −0.45], *N* = 40, *p* = 0.003) and this was checked and supported by our statistician.

Baldassarre et al. (2016) reported similar growth patterns between the two groups, according to body mass index at 4 weeks following treatment (time effect: *F* = 118.95, *p* < 0.001; treatment effect: *F* = 0.01, *p* = 0.92; interaction effect: *F* = 1.43, *p* = 0.24).

Garofoli et al. (2014) reported no difference in cranial circumference between the probiotic (37.33 cm, *SD*: 1.21) and placebo groups (38.03, *SD*: 1.47), *p* = 0.108 at 4 weeks.

### 3.5 Number of admissions to hospital, loss of parent working days, visits to any health professional related to infant regurgitation

Only one study (Indrio et al., 2014) found statistically significant less emergency department visits at 3 months in the probiotic group (MD: −1.26 visits, 95% CI: −1.43 to −1.09, *N* = 468, *p* = 0.00001). Indrio et al. (2014) also found statistically significant less paediatric visits due to the presence of symptoms in the probiotic group (MD: −1 visit, 95% CI: −1.12 to −0.88, *N* = 468, *p* = 0.00001). Indrio et al. (2014) found statistically significant fewer loss of parent working days in the probiotic group (MD: −2.35, 95% CI: −2.54 to 2.16, *N* = 468, *p* = 0.00001).

### 3.6 Admissions to hospital due to anxiety/depression

None of the included studies reported on admissions to hospital due to maternal anxiety/depression.

### 3.7 Adverse effects

None of the studies reported any adverse effects for the women or infants.

### 3.8 Risk of bias of included studies

The results of the 'Risk of bias' assessment for the included studies is summarised in Table 1.

**Table 1:** Characteristics of included trials

Study/year/reference	Description/study design	Age at enrolment/birth weight	Intervention probiotic agent(s)	Probiotic daily dose/(duration of intervention)	Control	No. analyzed/reported outcomes/results
Baldassarre/2016	*Timeline*: April 2011–Dec 2013.*Enrolments*: 67 healthy pregnant women (and matching infant), aged 18–44 years, admitted with low obstetric risk (infants were breastfed or breast and bottle fed after birth).*Setting*: Dept. of Biomedical and Human Oncological Science, University of Bari, ItalyStudy design: Double‐blind prospective randomised controlled trial	67 newborn term infants 37‐41 week's gestation. Birth weight 2440–4730 g	Probiotic administered to the women Four different strains of lactobacilli: *L. paracasei* DSM 24733, *L. plantarum* DSM 24730, *L. acidophilus* DSM 24735, *L. delbrueckii* subsp. *bulgaricus* DSM 24734. Three strains of bifidobacterial: *B. longum* DSM 24736, *B. breve* DSM 24732, *B. infantis* DSM 24737One strain of *Streptococcus thermophilus* DSM 24731	900 billion viable lyophilised bacteria in packets given to women.(36th week of pregnancy to 4 weeks after birth)	Placebo—Corn starch identical in sensory properties	*No. analyzed: N* = 66 women and infants (33 probiotics and 33 placebo)Power calculation based on outcome TGF‐β1 (27 probiotic and 27 placebo). *Infant regurgitation*: defined using the Rome III criteria. Outcome measured at 1 month of infants' life. Calculated as mean episodes/day over study period. Regurgitation more frequent in the placebo group vs. probiotic group (*χ* ^2^ = 6.944, *p* = 0.008; relative risk = 2.43(95% CI: 1.14–5.62)*.No. of stools/day*: No significant differences in number of bowel movements/day between the 2 groups (3.7 vs. 4.2, *t* = 1.17, *p* = 0.246). *Adverse events*: Nil reported.
Garofoli/2014	*Timeline*: not reported.*Enrolments*: 40 full term breastfed infants.*Setting*: Neonatal unit, Italy*.Study design*: Double‐blind prospective randomised controlled trial.	Infants 38.8–40.1 week's gestation. Infants enroled first 3 days of life. Birth weight 3243–3490 g.	*L. reuteri* DSM 17938 administered 5 drops. Not described.	1 × 10^8^ colony‐forming units in five drops once a day.(28 days)	Placebo —Administered as 5 drops. Not described.	No. analyzed: *N* = 40 infants (20 probiotics and 20 placebo)Power calculation not reported.*Infant regurgitation*: not defined. Outcome measured after 28 days of probiotic/placebo administration. Calculated as mean episodes/day over study period. Significant reduction in regurgitation for probiotic vs. placebo (*p* = 0.02). Other data provided as figures.*No. of stools/day*: No significant difference between the 2 groups (p value not provided).*Body weight*: No significant difference between the 2 groups (*p* = 0.53). *Body length*: No significant difference between the 2 groups (*p* = 0.087).*Cranial circumference*: No significant difference between the 2 groups (*p* = 0.108).*Adverse events*: Nil reported.
Indrio/2008	Timeline: Jan.–Sept. 2006*Enrolments*: 20 formula fed preterm infants with normal Apgar scores.*Setting*: Neonatology Section of the Dept. of Paediatrics, University of Bari, Italy.*Study design*: Double‐blind prospective randomised controlled pilot trial.	Preterm infants 34 ± 1.1 week's gestation. Enroled at 3 to 5 days of life. Birth weight 1890 ± 432 g.	*L. reuteri* ATCC 55730 administered in an oil formulation.	1 × 10^8^ colony‐forming units in 5 drops once a day in oil formulation.(30 days).	Placebo —Administered as 5 drops per day in an identical formulation	No. analyzed: *N* = 20 infants (10 probiotic and 10 placebo) Power calculation not reported. *Infant regurgitation*: defined as the passage of refluxed gastric contents into the oral pharynx. Outcome was measured after 30 days of probiotic/placebo administration. Calculated as mean episodes/day calculated over the last 7 days of treatment. Infants receiving probiotic showed a significant mean decrease in regurgitation 2.1 (*SD*: 0.9) vs. Placebo 4.2 (*SD*: 1.1), *p* < 0.01. *Gastric emptying time*: assessed by ultrasonography gastric emptying rate and calculated as % reduction in antral cross‐sectional area from time 0 to 120 min after meal ingestion. Gastric emptying rate was significantly faster in the probiotic group vs. placebo group (p = 0.001).*Number of stools per day*: Newborns receiving probiotics had a significant larger no. of stools 3.7 (*SD*: 0.5) compared to placebo 2.1 (*SD*: 0.4), *p* < 0.01. Daily weight gain (g): No difference in daily weight gain probiotics 28 (*SD*: 7.0) vs. placebo 25 (*SD*: 8.1). *Adverse events*: Nil reported.
Indrio/2011	Timeline: July 2008–Jan. 2010. Enrolments: 42 formula fed infants <4 months of age with diagnosis of uncomplicated regurgitation were enroled.Setting: Gastrointestinal Unit of the Dept. of Paediatrics at the University of Bari, Italy. Study design: Double‐blind prospective randomised controlled trial.	Term infants (gestation not provided). Age in days at enrolment: 31–45 days. Weight at enrolment: 4990–5100 g.	*L. reuteri* DSM 17938 suspended in a mixture of pharma grade sunflower and medium‐chain triglyceride oils.	1 × 10^8^ colony‐forming units in 5 drops once a day.(30 days).	Placebo —Identical formulation in all respects except that the live bacteria were excluded. No differences smell or taste.	No. analyzed: *N* = 34 (19 probiotics and 15 placebo).Power calculation based on outcome gastric emptying (11 infants per group).*Infant regurgitation*: defined according to the ROME III criteria. Outcome measured after receiving the probiotic/placebo for 4 weeks. Calculated as median episodes/day calculated over the last 7 days of treatment. Median episodes per day of regurgitation was reduced in probiotic group 1.0 (1.0–2.0) vs. placebo group 4.0 (3.0–5.0), *p* < 0.001).*Gastric emptying rate*:Calculated as % reduction in antral cross‐sectional area at time 0 and 120 min after meal ingestion. The change in gastric emptying rate (the difference in the % GErate values before and after intervention) was then calculated. Change in gastric emptying rate was significantly increased in infants receiving probiotics 11.7 (3.9 to 24)% compared to placebo vs. 8.4 (27.0 to 23.5)%, *p* = 0.01.*Body weight and growth*: 'No difference in body weight and other growth parameters the end of the trial.'*Adverse events*: No adverse events
Indrio/2014	Timeline: Sept. 2010–Oct. 2012.Enrolments: 554 breast or formula fed term neonates <1 week of age.Setting: 9 different neonatal units, Italy.Study design: Double‐blind prospective randomised controlled trial.	Gestation: 37–41 weeks. Age <1 week at enrolment. Birth Weight: probiotic Mean 3378 (*SD*: 413) Placebo Mean 3302 (*SD*: 392).	*L reuteri* DSM 17938	1 × 10^8^ colony‐forming units in 5 drops. Probiotic‐mixture of pharmaceutical grade sunflower and medium‐chain triglyceride oils.(90 days)	Placebo —Consisted of an identical formulation of oils, except that the live bacteria were excluded. No differences in smell or taste	No. analyzed: *N* = 468 (Probiotic group: 238; 38 lost to follow up; placebo group: 230; 48 lost to follow up = 68%). Sample size calculation based on outcome regurgitation (230 in each group).*Regurgitation*: defined as the passage of refluxed gastric contents into the oral pharynxOutcomes measured at 1 and 3 months after commencement of probiotic/placebo. Calculated as mean episodes/day over study period. No significant difference in mean regurgitation for probiotic group 2.7 (SD: 1.5; CI: 2.5–2.9) and the placebo group 3.3 (*SD*: 2.3; CI: 3.0–3.6), *p* = 0.35 after 1 month of intervention. Mean number of regurgitations per day was reduced in probiotic group 2.9 (*SD*: 1.1; CI: 2.7–3.0) vs. placebo group 4.6 (*SD*: 3.2; CI: 4.2–5.0); *p* < 0.01 after 3 months of intervention.*No. stools/day*: Mean no. stools/day after 1‐month intervention: probiotic 4.01 (*SD*: 1.1) vs. placebo 3.3 (*SD*: 2.3); *p* < 0.01. Mean no. stools/day after 3 months intervention: probiotic 4.2 (*SD*: 1.8) vs. placebo 3.6 (*SD*: 1.8); *p* < 0.01. Emergency dept. visits after 3 months intervention: Probiotics 0.52 (0.72) vs. placebo 1.78 (1.11). *Paediatric visits* after 3 months intervention: Probiotic 1.3 (*SD*: 0.6) vs. placebo 2.3 (*SD*: 0.7), *p* < 0.05. Mean loss of parent working days after 3 months intervention: probiotic 0.54 (*SD*: 0.62) vs. placebo 2.89 (*SD*: 1.3), *p* < 0.05.*Adverse events*: No adverse events reported.
Indrio/2017	Timeline: Jan. 2014–Feb. 2015. Enrolments: 80 exclusively formula fed full‐term infants diagnosed with functional regurgitation were enroled. Setting: Paediatric Gastroenterology Clinic, Dept. of Paediatrics) of the University of Bari, Italy), and a Paediatric Primary Care Clinic in Naples, Italy. Study design: Double‐blind prospective randomised trial	Term infants (gestation not provided) aged 4 weeks–5 months at enrolment (average 60 days of age). Weight at enrolment: Probiotic—5590 ± 631. Placebo—5670 ± 739	*L. reuteri* DSM 17938 and commercially available partially hydrolysed 100% whey formula thickened with starch, providing 1.9 g protein per 100 kcal, and supplemented with a mixture of potato, corn starch (4 g/100 kcal) (test formula)	2.8 × 10^6^ colony‐forming units/g powder for 4 weeks	Control —Commercially available starter formula that included 70% whey protein and 30% casein, providing 1.85 g of protein per 100 kcal	No. analyzed: *N* = 72 (37 probiotic and 35 placebo).The sample size calculation based on gastric emptying rate (30 infants per group).*Regurgitation*: Defined according to the Rome III criteria based on retrospective reports from parents. Outcome was measured after receiving the probiotic/placebo for 4 weeks. Calculated as mean episodes over the last 7 days of treatment. Mean number of regurgitations per day reduced for test formula + probiotic 2.6 (*SD*: 1.0; 95% CI: 2.2–2.9) vs. control 5.3 (*SD*: 1.0; 95% CI: 5.0‐5.6), *p* < 0.0001.*Gastric emptying rate*: Calculated as percent reduction in antral cross‐sectional area at time 0 and 120 min after meal ingestion then percentage difference between baseline GErate values and values at end of week 4. (GErate % change from week 0 to week 4)Test formula showed a significantly higher GErate percentage 12.3 (−3.9, 22.0) vs. control 9.1 (−27.0, 25.5), *p* < 0.021.Adverse events: Nil adverse events.

#### 3.8.1 Random sequence generation

All six studies described an adequate method of random allocation of participants to intervention groups, so the studies were rated low risk of bias.

#### 3.8.2 Allocation concealment

All six studies described an adequate allocation concealment of participants and personnel, so the studies were rated low risk of bias.

#### 3.8.3 Blinding of participants and personnel

All six studies reported blinding of participants and personnel.

#### 3.8.4 Blinding of outcome assessment

All six studies were rated low risk of bias for blinding of outcome assessment.

#### 3.8.5 Incomplete outcome data

We rated all six studies as low risk of attrition as dropouts were low and balanced across the treatment groups.

#### 3.8.6 Selective reporting

Two studies were rated at high risk of reporting bias (Baldassarre et al., 2016; Indrio et al., 2017). Baldassarre et al. (2016) did not identify the outcome regurgitation in the trial registration record but reported regurgitation as a secondary outcome in the published article. Indrio et al. (2017) reported the outcome of regurgitation (difference in the proportion of improved subjects at 4 week of treatment) as the primary outcome, and regurgitation score: severity of regurgitation, frequency of regurgitation, volume of regurgitation in the trial registration record. However, only regurgitation (frequency of regurgitation episodes) was reported as a secondary outcome in the published article.

The remaining four studies were rated unclear risk of reporting bias. We were not able to locate a trial registration record for Indrio et al. (2011, 2008), and Garofoli et al. (2014). One study (Indrio et al., 2014) was rated as unclear risk of reporting bias because the outcome regurgitation was measured at 1 and 3 months in the published report, but the trial registration record reported the outcome to be measured only at 3 months. Indrio et al. (2011) reports the findings for the outcome regurgitation as medians and 5 and 95 percentiles, however, the other studies by Indrio are reported as mean and *SD*. Indrio et al. (2014) reported a nonsignificant result for the outcome regurgitation after 1 month of treatment (*p* = 0.35), however, we repeated this analysis and found a significant difference between probiotics and the control group (*p* = 0.0009). Garofoli et al. (2014) reported a nonsignificant difference for the outcome length of baby after 1 month of treatment (*p* = 0.087), however, we calculated the summary data and found there to be a significant difference between the probiotic and control groups (*p* = 0.003). All studies reported on adverse events.

#### 3.8.7 Other potential sources of bias

We considered the five studies that were supported by the manufacturer of the intervention to be at high risk of bias (Baldassarre et al., 2016; Garofoli et al., 2014; Indrio et al., 2011, 2014, 2017). Indrio et al. (2017) reports that the infant formula (that contained the probiotic) was supplied by the manufacturer and provided no other funding and had no role in the design of the study. The chief investigator served as a speaker for the Nestle Nutrition Institute. Indrio et al. (2014, 2011) report that the study was partially supported by the manufacturer of the probiotics and had no role in other aspects of the study. Garofoli et al. (2014) reports that the probiotics and placebo were supplied by the manufacturer, no other information is provided. Baldassaree et al. (2016) reports that the probiotics were gifted from an individual (who developed the eight‐strain cocktail of antibiotics). All studies were undertaken in Italy, and five of the six studies had the same chief, or coinvestigator. Indrio et al. (2008) does not report on any support received from the manufacturer of the probiotics and is rated as unclear risk of bias.

## 4 DISCUSSION

To our knowledge, we report the first systematic review to investigate the efficacy of probiotic supplementation for the prevention and treatment of infant regurgitation. It involved a rigorous review process with adherence to internationally recognised Cochrane and Preferred Reporting Items for Systematic Reviews and Meta‐analyses guidelines. We found six RCTs on the use of probiotics for the prevention or treatment of infant regurgitation for inclusion in the systematic review.

Meta‐analysis of three of the six trials showed a statistically significant reduction in regurgitation in the infants receiving *L. reuteri* DSM 17938 (Indrio et al., 2014, 2017) or the original strain, *L. reuteri* ATCC 55730 (Indrio et al., 2008) probiotic compared to the placebo. There was high heterogeneity between the studies that was most likely due to the heterogeneity of the participants; for example, age at time of enrolment, type of feeding (bottle/breast), gestation (preterm/term), and pre‐existent/nonexistent regurgitation and dosage of the probiotic.

The remaining individual studies also reported a statistical reduction in episodes of regurgitation with the use of probiotics (Garofoli et al., 2014). Only one small study examined maternal probiotic use in the antenatal and postnatal periods and showed a significant reduction in the onset of infant regurgitation (Baldassarre et al., 2016). While there was an overall low risk of bias in the conduct of the studies, there was substantial heterogeneity between the trials and the results need to be viewed with caution. Several in vitro studies have proven that *L. reuteri* is also found to exhibit antimicrobial activity, producing reuterin, a broad‐spectrum antibacterial substance (Axelsson et al., 1989; Talarico et al., 1988) and regulate immune responses (Lin et al., 2008) as well as reduce intestinal inflammation (Liu et al., 2010); thus it is possible that *L. reuteri* strains act through diverse mechanisms.

Individual studies found that gastric emptying was statistically significantly faster in the infants receiving probiotics compared with placebo. It has been reported that probiotics improve gut motility and gastric emptying time and thus reduces gastric distension and visceral pain (Garofoli et al., 2014; Wang et al., 2010). Meta‐analysis of two studies using *L. reuteri* ATCC 55730 and *L. reuteri* DSM 17938 found a statistically significant increase in the number of stool evacuations and it has reported that probiotics could play a crucial role in the modulation of intestinal inflammation that may contribute to infant regurgitation (Indrio et al., 2014), but there was moderate heterogeneity. It does not appear that probiotics have a positive or negative effect on infant body weight, head circumference, or length. There appear to be no safety concerns with the administration of probiotics. Only one study (Indrio et al., 2014) reported on number of hospital admissions, loss of parent working days, and visits to a paediatrician and found these outcomes were significantly statistically lower in the probiotic group compared to the placebo group.

The impact of infant immaturity, disturbance of the microbiome through caesarean section and maternal mental health has been recently considered. A mixed methods study examined greater than 1 million admissions of infants in NSW, Australia to hospitals in the first year following birth (Dahlen et al., 2018). In addition, the records of greater than 11,000 babies admitted with infant regurgitation were examined. Infants with regurgitation admitted to hospital were also likely to have other disorders such as feeding difficulties, sleep problems, and excessive crying. The mothers of babies admitted with regurgitation were more likely to be primiparous, Australian born, give birth in a private hospital and have a psychiatric condition. In addition, the mothers were more likely to have a preterm or early term infant (37–38 weeks), a caesarean section, an admission of the baby to a SCN/NICU and be a male infant (Dahlen et al., 2018). The records of 300 women and babies admitted to residential parenting services in NSW (RPS) were also randomly examined in the study by Dahlen et al. (2018) and found 36% of infants admitted to residential parenting centres in NSW had been given a diagnosis of infant regurgitation (Priddis et al., 2018). Eight focus groups were undertaken with 45 nurses and doctors working in these RPS and the qualitative data revealed two themes: 'It is over diagnosed' and 'A medical label is a quick fix, but what else could be going on?' (Dahlen et al., 2018).

None of the included studies in this systematic review reported on admissions to hospital due to maternal anxiety/depression. The study by Dahlen et al. (2018) also found that mothers with a mental health disorder were nearly five times as likely to have a baby admitted with regurgitation in the first year after birth. This finding is significant and needs further exploration as to the possible mechanism and possible prevention/treatment. It is possible that inconsistent parenting by inexperienced and anxious mothers may increase infant crying. The fact that primiparous women were more likely to have an infant with regurgitation supported this (Dahlen et al., 2018). However, it is possible that maternal mental health has a bidirectional relationship with a disturbed microbiome in the mother and the baby. This is where we propose there may be a role for probiotics in restoring a balance and thereby impacting on severity of regurgitation symptoms. Probiotic administration antenatally, and for the first few months following birth, may also have a modifying effect on maternal mental health by modifying the microbiome and thus impacting on the brain/gut axis. This is particularly effective with stress‐related psychopathologies such as anxiety and depression. We recommend that future studies examining the use of probiotics for infant regurgitation should also examine maternal anxiety and depression.

Of the six studies included in the review, five administered *L. reuteri* DSM 17938 or its original strain, *L. reuteri* ATCC 55730 (Garofoli et al., 2014; Indrio et al., 2014; Indrio et al., 2011, 2017). When trying to evaluate why these strains were used, we found that each study based their choice on the good results of a previous study. These studies found the following positive results for *L. reuteri* administration: increased gastric emptying and reduction in crying time, regurgitation episodes, constipation, and fasting antral area. The authors based their findings on changes in intestinal microbiota, improved mucosal barrier, anti‐inflammation, improved motility of the whole intestine and neuroimmune interaction. However, other strains of probiotics could also perform such actions.

### 4.1 Quality of the evidence

**Figure 5:**
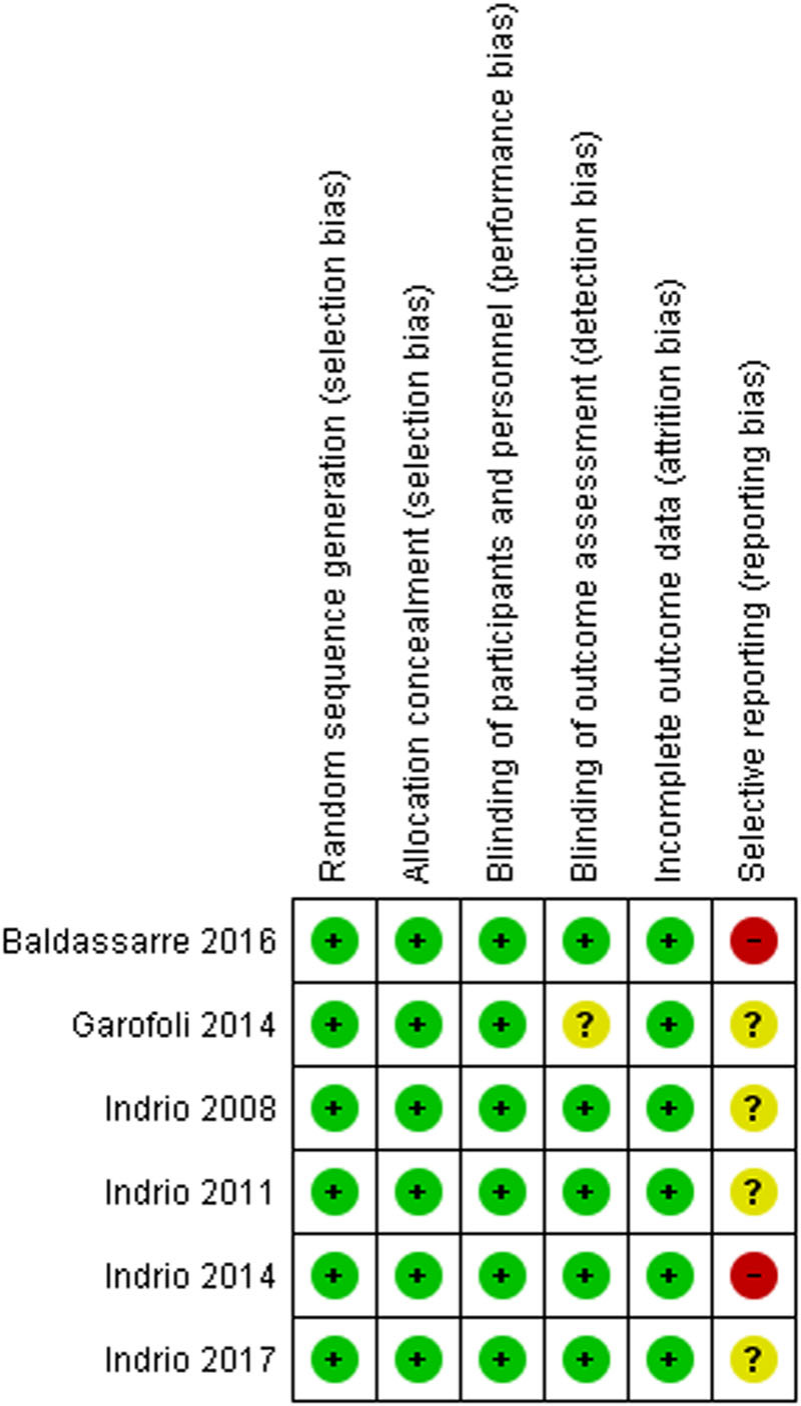
Risk of bias in the trials

We thoroughly reviewed the studies for results and assessed their risks of bias. There was an overall low risk of bias in the trials for random sequence generation, allocation concealment, blinding of participants and personnel, blinding of outcome assessment and incomplete outcome data. There was an overall high risk or unclear risk of selective reporting bias and five of the six trials reported receiving financial support from the manufacturer or the makers of the probiotic used (Figure 5).

## 5 CONCLUSIONS

The currently available evidence does not support or refute the efficacy of probiotics for the prevention and treatment of infant regurgitation. However, data from the individual trials and subset meta‐analysis of studies measuring the effect of probiotics are promising. There are no indications from the available data that probiotics have any adverse effects. The use of probiotics could potentially be a noninvasive, safe, cost effective and preventative positive health management strategy for both women and their babies. Further well‐controlled RCTs are warranted to investigate the efficacy of various strains and species, dosage, and combinations of probiotics to determine the most effective for preventing and treating infant regurgitation. In addition, further research is required to determine the effectiveness of administering probiotics to women antenatally and/or postnatally to prevent regurgitation in breastfed infants and their effect on maternal mental health.

## ACKNOWLEDGEMENTS

This project was supported by the School of Nursing and Midwifery, Western Sydney University.

## CONFLICT OF INTERESTS

The authors declare no conflict of interests.

## AUTHOR CONTRIBUTIONS

Jann P. Foster, Charlene Thornton, and Hannah G. Dahlen conceived the research project and coordinated the contributors. Jann P. Foster and Kim Psaila developed the search strategy and searched the databases. Jann P. Foster, Kim Psaila, and Hannah G. Dahlen participated in the study selection. Jann P. Foster, Kim Psaila, and Hannah G. Dahlen performed the data extraction. Jann P. Foster, Kim Psaila, Hannah G. Dahlen, and Sabina Fijan interpreted the findings. Jann P. Foster, Hannah G. Dahlen, Kim Psaila, and Sabina Fijan wrote the first draft of the results and Nadia Badawi, Virginia Schmied, Charlene Thornton, and Caroline Smith revised subsequent drafts. Jann P. Foster, Hannah G. Dahlen, Kim Psaila, and Sabina Fijan prepared the manuscript and revised every version of the manuscript. Nadia Badawi, Virginia Schmied, Charlene Thornton, and Caroline Smith contributed to the revision of the manuscript.

## Data Availability

As this article is a systematic review, the data that support the findings are articles published in academic journals that are already in the public domain. Therefore, data sharing is not applicable to this article as no new data were created or analyzed.
